# Ethnology of the Negro or Prognathous Race

**Published:** 1858-04

**Authors:** Sam’L A. Cartwright


### SELECTIONS.

*Ethnology of the Negro or Prognathous Race. A Lecture delivered Nov. 30, 1857 before the New Orleans Academy of Sciences.* By SAM'L A. CARTWRIGHT, M. D.

The natural history of mankind is divided into two parts—Ethnography and Ethnology. The former will be passed over, as it treats of the physical difference among men, their geographical distribution, history, origin, ect; and the latter only will be considered, or that science which investigates the moral and mental differences among the different groups of mankind, and searches for the law on which they depend. There are three principal groups, each of which has maintained the physical traits and mental characteristics peculiar to it, unaltered by time, circumstances or adventitious causes, for a period as far back as history extends. Hence they are called primordial, or primitive. Natural historians designate them, as the white, yellow and black: otherwise, the Indo-European, Mongolian and Prognathous.

The ethnology of the Prognathous or negro race, is the subject under consideration.

When we look around us, we behold four millions of that group of mankind engaged mostly in agriculture, and under subjection to that other group, called Indo-European, Indo-Germanic, Arien, Caucasian, or white. Ethnology is interrogated, to know if this be their *normal condition?* It answers in the affirmative, and its reasons are called for. The question has been answered variously, by religionists and politicians, which has given rise to much angry disputations, recriminations, costly experiments, useless restrictions, senseless alarms and disquietude—all of which might have been prevented, if the subject had been examined as an ethnological one.

Every one of the three primordial groups of mankind, and the subdivisions of those groups into tribes and nations, have their variables instincts, appetites, likes and dislikes, habits, customs, intellectual capacities, moral standards, religious sentiments, ect., distinguishing one from the other, all of which have to be carefully observed through a long series of years before those elementary principles can be reached on which the science of ethnology rests as a basis.

George III. had not studied the ethnology of his colonial subjects in America, or, it would have told him that it was impossible to bend their necks. The flames of Moscow taught Napoleon the ethnical truth, that Russians differ in instincts and habits of thought from Frenchmen—as he knew that nothing would tempt Frenchmen to burn Paris.

The English, Irish and Scotch, have very perceptible shades of difference in character. The whole trio differ essentially from their neighbors across the channel. Ten millions of them, the *bourgeoise*, at the head of civilized nations, differ essentially from the twenty-six millions of unim provable peasantry, who are even behind the Hindoos and Chinese, as few of them can read or write; whereas, there is scarcely an inhabitant of Hindostan or the Chinese empire who cannot read a book. There is something inherent in the French, which enables them to resist the diseases of this latitude better than most other Europeans, or our own people, of the North and West. The inefficient tisane practice in acute diseases, so generally adopted by them, is a fearful trifling with life when imitated in the treatment of the acute diseases of the Englishman, German or American. Neither the same systems of government nor the same systems of medical practice are alike applicable to all nations and races of people.

Ethnology, therefore, disowns a common standard formula in government, civilization, morals and medicine, as alike applicable to all men.—Where the ethnical elements are different, a common formula is empirical. Diversity of locality as well as diversity of races, should also be regarded. The yellow fever is more obedient to remedial agents in New Orleans than in localities further north. A negro never dies with it in any locality, when treated with regard to his ethnical peculiarities. So strong are they in his favor that, even under mal-practice, death is the exception and recovery, the rule. A number of the prognathous race died in the epidemic of New Orleans, 1853, more from panic, it is believed, than from the yellow fever of that year. Experience and observation prove that panic is very apt to kill a negro, but it is questionable whether the yellow fever *per se* has that power. So seldom does it attack individuals of that race, that Dr. Rush was disposed to regard the negroes as black angels, sent by a kind Povidence to nurse the sick during the terrible yellow fever which ravaged our Northern cities for a number of years in succession, about the close of the last century and beginning of the present. Much abolition capital was made out of the idea that this exemption was due to a special Providence, conferring on them a special opportunity to display their gratitude to those who had advocated the doctrine of their equality. Not only the great Rush, but many other good and distinguished men have fallen into grave mistakes in regard to the prognathous race of mankind, in consequence of not setting in the light of ethnological science. That science proves that the moral and intellectual diversities between the prognathous and Indo-European races, are actually greater than their physical. These diversities have continued as far back as history extends, unalterably the same in every climate and under every variety of circumstances, short of amalgamation. In that case, the offspring is a *tertium quid*, unlike either father or mother, and incapable of perpetuating it existence beyond a few generations. Dr. Day, of the Winnebago agency, avers, that the offspring of the Indian and negro has so little vitality that it ceases to exist after the fourth generation. The Mexicans, a mixture of the Indian, negro and white races, are dying out. The hybrid, from a mixture of the negro with the dark skinned European nations, has more vitality than the Irish, English or American hybrid.

Numbers definite in value reign supreme, unaltered and unalterable, throughout the vast domains of inorganic nature. All the simple elements have separate combining numbers peculiar to them. The combining number of oxygen never varies and never can. Its potential equivalent is always the same. It is the only element capable of forming combinations with all the other elements. Hydrogen is an element, which has not the same capacity for combination as oxygen. It is just as impossible to elevate hydrogen in its combining capacities, as to degrade oxygen. They differ in their potential equivalents as one to eight. It is beyond the power of man, or the chemist's art, to make them equal. If politicians or religionists should take it in their heads to do so, on some *a priori* reasoning, that being elements at the base of the inorganic kingdom they ought to be equal in potency; and that it was only for the want of well-directed experiments that the latent capacities of hydrogen remained concealed; the chemist could tell them that they might waste millions on such experiments, or destroy empires, to elevate one or depress the other, and they would be no nearer bringing them to a level than when they first began. So, also in the organic kingdom, it is just as impossible to add to or subtract from the inherent potency that nature has measured out to the different types of mankind. The prognathous type of man, after all the costly experiments that have been made to elevate him, remains the same that he was and ever will be in inherent potency, when compared to the Indo-European. Like an inferior planet, he can be drawn out of his natural orbit by a race of superior potency to his own, but sinks into his original status again as soon as he ceases to be acted on and sustained by the exterior power that elevated him out of it. All history proves, that when left to itself the prognathous race has never originated a civilization of its own, or sustained one that had been given to it.

The facts, gathered from all quarters of the extensive domain of ethnological science, have been compared and generalized. They are harmonious in declaring that each of the three great groups of mankind has its own special mode of development, its own special potency and series of expansions into a result, beyond which it cannot pass; that result being the termination of its progress, expresses the realization of the end and object of its creation. The ancient Greeks, in oratory, poetry, painting and sculpture, progressed by successive unfoldings until they arrived at an ultimatum, beyond which the Indo-European race cannot go—its inherent special potency having stretched itself to its utmost tension. The Chinese reached the ultimatum of progress that the Mongolian race is capable of attaining. That progress stopped far short of the point the Greeks reached. The ultimate limit of progress the prognathous race has ever made, stops within the confines of barbarism. There its inherent vital potency gives out and can carry it no further. While the yellow types progressed beyond, but came to a stand-still in the fields lying between barbarism and civilization. None but the white type has ever forced its way and maintained its position in that high order of civilization where moral virtue, clad in intellectual light, rules society. The yellow types, or the Arabs, for instance, in the Medieval ages, have often by adventitious circumstances, been brought within the realm of intellectual and moral light, but have invariably fallen back again into the twilight of semi-civilization, their instincts rebelling against the restraints which the moral influence of that higher order of civilization, whose woman is regarded as the better half of man, imposed on them. Their instincts dragged them down to that lower order of civilization, (*if it*

*can be called civilization,)* where the female is regarded as an inferior being and treated as a slave. The Indo-European, or white man, whether in the civilized or savage state, has always instinctively regarded and treated woman as a companion more worthy and entitled to more respect than himself. Cæsar was so well apprised of this ethnical fact that in treaties made with white savages (the Germans) he demanded their women for hostages, well knowing that they put a higher value upon them than upon their most renowned warriors. It is all a mistake about civilization giving liberty to woman, or elevating her in man's estimation. He owes his civilization to her; because he begins to lose it when out of her presence or cut off from her society. So far from any of the yellow or black races, tribes or nations, regarding her as a divinity or as a companion, they all look upon her as a being of an inferior caste, and enslave her. The Chinese, the highest among them, deny to woman the right to choose husbands; the husband's power is unlimited over the wife. She is not only his slave, but is bound to obey every member of his family—his brothers, sisters, uncles, and aunts. The prognathous Australians obtain their wives by knocking them down with a club and wounding them in the foot to prevent them from running away. The Nigritians, or Africans proper, not only enslave their wives, but pave their court-yards with the skulls of the refractory or disobedient. Wealth and power acquired by the husband, so far from elevating the wives, add to their degredation by being used to increase their number. The king of Dahomey, according to the most authentic accounts, has 3333 living wives, besides a vast number whom he has capriciously murdered. Nor does, what is called negro freedom, elevate the colored women, but sinks them lower. The husband holds them in abject slavery; they dare not kill them, as in Africa, but they beat and maltreat them in the cellars of New York, and other places in the Northern States, which they dare not do in the South. The freedom of the husband, is a loss to the women and children. They are in slavery still, and have lost their white protectors. While they and their husbands were in subordination to the white man, equal rights and equality among all the colored population, male and female, young and old, are sedulously maintained, as indispensable on every Southern plantation; all of which is lost to three-fourths of the black population, the women and children, by what is called negro freedom. Nor are the negro men themselves free in anything but name A late celebrated ethnologist; De Gobineau, adduces Hayti as a glaring instance of the futility of the attempt to give a people institutions not suggested by their own genius or instincts. He says, "that there, as in Africa, the negro drinks ardent spirits, butchers his enemies, propitiates his sorcerers, and the rest of the time sleeps."

Under all the appliances of British power to prevent it, the prognathous race of the British possessions on this continent are going the same way as in Hayti. The colonies at Liberia and Sierre Leone would long since have been lost among savages, but for the almost superhuman exertions of the missionaries, and the fortunate aid derived from the apprenticeship system, subjecting the wild Africans to a species of slavery to indolent colonists.

The Nilotic monuments furnish numerous portraits of the negro races, represented as slaves, sixteen hundred years before the Christian era.—Although repeatedly drawn from their native barbarism and carried among civilized nations, soon forget what they learn and relapse into barbarism. If the inherent potency of the prognathous type of mankind had been greater than it actually is, sufficiently great to give it the independence of character that the American Indians possess, the world would have been in a great measure deprived of cotton and sugar. The red man is unavailable as a laborer in the cane or cotton field, or any where else, owing to the unalterable ethnical laws of his character. The white man cannot endure toil under the burning sun of the cane and cotton field, and live to enjoy the fruits of his labor. The African will starve rather than engage in a regular system of agricultural labor, unless impelled by the stronger will of the white man. When thus impelled, experience proves that he is much happier, during the hours of labor in the sunny fields, than when dozing in his native woods and jungles. He is also eminently qualified for a number of employments, which the instincts of the white regard as degrading. If the white man be forced by necessity into employments abhorrent to his instincts, it tends to weaken or destroy that sentiment or principle of honor or duty, which is the mainspring of heroic action from the beginning of historical times to the present, and is the basis of everything great and noble in all grades of white society.—The importance of those particular employments, regarded as servile and degrading by the white man, attended to by the black race, whose instincts are not repugnant to them, will be at once apparent to all those who deem the sentiment of honor or duty as worth cultivating in the human breast. It is utterly unkown to the prognathous race of mankind, and has no place in their language. When the language is given to them they cannot comprehend its meaning, or form a conception of what is meant by it. Every white man, who has not been degraded, had rather be engaged in the most laborious employments, than to seem as a lacquey or body-servant to another white man, or being like himself. Whereas, there is no office, which the negro or mulatto covets more than that of being a body servant to a real gentleman. There is no office which gives him such a high opinion of himself, and it is utterly impossible for him to attach the idea of degradation to it. Those identical offices which the white man instinctively abhors, are the most greedily sought for by negroes and mulattoes, whether slave or free, in preference to all other employments. North or South, free or slave, they are ever at the elbow, behind the table, in hotels and steamboats; ever ready, with brush in hand, to brush the coat or black the shoes, or to perform any menial service which may be required, and to hold out the open palm for the dime. The innate love to act as body-servant or lacquey, is too strongly developed in the negro race to be concealed. It admirably qualifies them for waiters and house-servants, as their strong muscles, hardy frames, and the positive pleasure that labor in a hot sun confers on them, abundantly qualify them for agricultural employments in a hot climate.

Hence the primordial cell-germ of the Nigritian has no more potency than what is sufficient to form a being with physical power, when its dynamism becomes exhausted, dropping the creature in the wilderness with the mental organization too imperfect to enable him to extricate himself from barbarism. If Nature had intended the prognathous race for barbarians as the end and object of their creation, they would have been like lions and tigers, fierce and untamable. So far from being like ferocious beasts, they are endowed with a will so weak, passions so easily subdued, and dispositions so gentle and affectionate, as readily to fall under subjection to the wild Arab, or any other race of men. Hence they are led about in gangs of an hundred or more by a single individual, even by an old man, or a cripple, if he be of the white race and possessed of a strong will. The Nigritian has such little command over his own muscles, from the weakness of his will, as almost to starve, when a little caution and forethought would procure him an abundance. Although he has exaggerated appetites and exaggerated senses, calling loudly for their gratification, his will is too weak to command his muscles to engage in such kinds of labor as would readily produce the fruits to gratify them. Like an animal in a state of hibernation, waiting for the external aid of spring to warm it into life and power, so does the negro continue to doze out a vegeto-animal existence in the wilderness, unable to extricate himself therefrom—his own will being too feeble to call forth the requisite muscular exertion. His muscles not being exercised, the respiration is imperfect, and the blood is imperfectly vitalized. Torpidity of body and hebetude of mind are the effects thereof, which disappear under bodily labor, because that expands the lungs, vitalizes the blood and wakes him up to a sense of pleasure and happiness unknown to him in the vegeto-animal or hibernating state Nothing but will is wanting to transform the torpid, unhappy tenant of the wilderness into a rational and happy being—the happiest being on earth, as far as sensual pleasures are concerned. The white man has an exaggerated will—more than he has use for; because it frequently drives his own muscles beyond their physical capacity of endurance. The will is not a faculty confined within the periphery of the body. It cannot, like the imagination, travel to immeasurable distance from the body, and in an instant of time go and return from Aldabaran, or beyond the boundaries of the solar system. Its flight is confined to the world and to limits more or less restricted—less restricted in some than in others. The will has two powers—direct and indirect. It is the direct motor power of the muscular system. It indirectly exerts a dynamic force upon surrounding objects when associated with knowledge. It gives to knowledge its powers. Everything that is made was made by the Infinite Will associated with Infinite knowledge. The will of man is but a spark of the Infinite Will, and its power is only circumscribed by his knowledge. A man possessing a knowledge of the negro character can govern an hundred, a thousand or ten thousand of the prognathous race by his will alone, easier than one ignorant of that character can govern a single individual of that race by the whip or a club. However disinclined to labor the negroes may be, they cannot help themselves; they are obliged to move and to exercise their muscles when the white man, acquainted with their character, *wills* that they should do so. They cannot resist that will, so far as labor of body is concerned. If they resist, it is from some other cause than that connected with their daily labor. They have an instinctive feeling of obedience to the stronger will of the white man, requiring nothing more than moderate labor. So far, their instincts compel obedience to his will as one of his rights. Beyond that, they will resist his will and be refractory, if he encroaches on what they regard as their rights, viz., the right to hold property in him as he does in them, and to disburse that property to them in the shape of meat, bread and vegetables, clothing, fuel and house room, and attention to their comforts when sick, old, infirm and unable to labor; to hold property in him as a conservator of the peace among themselves, and a protector against trespassers from abroad, whether black or white; to hold property in him as an impartial judge and an honest jury to try them for offences, and a merciful executioner to punish them for violations of the laws or usages of the plantation or locality. With those rights conceded to them, no other compulsion is necessary to make them perform their daily tasks than *his will alone.* It is not the whip, as many suppose, which calls forth those muscular exertions, the result of which is sugar, cotton, breadstuffs, rice and tobacco. These are products of the white man's will acting through the muscles of the prognathous race in our Southern States. If that will were withdrawn, and the plantations handed over as a gracious gift to the laborers, agricultural labor would cease for the want of that spiritual power, called the will, to move these machines—the muscles. They would cease to move here, as they have in Hayti. If the prognathous race were expelled from the land, and their place supplied with double their number of white men, agricultural labor in the South would also cease, as far as sugar and cotton are concerned, for the want of muscles that could endure exercise in the smothering heat of a cane or cotton field. Half the white laborers of Illinois are prostrated with fevers form a few days' work in stripping blades in a Northern cornfield, owing to the confinement of the air by the close proximity of the plants. Cane and cotton plants form a denser foliage than corn; a thick jungle, where the white man pants for breath, and is overpowered by the heat of the sun at one time of day, and chilled by the dews and moisture of the plants at another. Negroes glory in a close, hot atmosphere; they instinctively cover their heads and faces with a blanket at night, and prefer lying with their heads to the fire, instead of their feet. This ethnical peculiarity is in harmony with their efficiency as laborers in a hot, damp, close, suffocating atmosphere—where, instead of suffering, and dying, as the white man would, they are healthier, happier and more prolific than in their native Africa—producing, under the white man's will, a great variety of agricultural products, besides upwards of three millions of bales of cotton, and three hundred thousand hogsheads of sugar. Thus proving that subjection to his will is normal to them, because, under the influence of his will, they enjoy life more than in any other condition, rapidly increase in numbers, and steadily rise in the scale of humanity.

The power of a stronger will over a weaker, or the power of one living creature to act on and influence another, is an ordinance of Nature which has its parallel in the inorganic kingdom, where pondorous bodies, widely separated in space, influence one another so much as to keep up a constant interplay of action and re-action throughout Nature's vast realms. The same ordinance, which keeps the spheres in their orbits and hold the satellites in subordination to the planets, is the ordinance that subjects the negro race to the empire of the white man's will From that ordinance the snake derives its power to charm the bird, and the magician his power to amuse the curious, to astonish the vulgar, and to confound the wisdom of the wise. Under that ordinance, our four millions of negroes are as unalterably bound to obey the white man's will, as the four satallites of Jupiter, the superior magnetism of that planet. Individual masters, by releasing individual negroes from the power of their will, cannot make them free or release them from subordination to the instinctive public sentiment or will of the aggregate white population, which as rigidly excludes them, in the so-called free States, from the drawing room and parlor as it does pots and kettles and other kinds of kitchen furniture. The subjugation of equals to equals by artifice or force is tyranny or slavery; but there is no such thing in the United States, because equals are on a perfect equality here. The subordination of the Nigritian to the Caucasian would never have been imagined to be a condition similar to European slavery, if any regard had been paid to ethnology. Subordination of the inferior race to the superior is a normal, and not a forced condition. Chains and standing armies are the implements used to force the obedience of equals to equals—of one white man to another. Whereas, the obedience of the Nigritian to the Caucasian is *spontaneous*, because it is normal for the weaker will to yield obedience to the stronger. The ordinance which subjects the negro to the empire of the white man's will, was plainly written on the heavens during our Revolutionary war. It was then that the power of the united will of the American people rose to its highest degree of intensity. Every colony was a slave holding colony excepting one; yet the people, particularly that portion of them residing in districts where the black population was greatest, hastened to meet in the battle-field the powerful British armies in front of them, and the interminable hosts of Indian warriors in the wilderness behind them, leaving their wives and children, their old men and cripples, for seven long years, *to their negroes to take care of*. Did the slaves, many of whom were savages recently imported from Africa, butcher them, as white or Indian slaves surely would have done, and fly to the enemy's standard for the liberty, land, money, rum savage luxuries, and ample protection so abundantly promised and secured to all who would desert their master's families? History answers that not one in a thousand joined their master's enemies, but on the contrary, they continued quietly their daily labors, even in those districts where they outnumbered the white population ten to one. They not only produced sufficient breadstuffs to supply the families of their masters, but a surplus of flour, pork and beef was sent up from the slave-holding districts of Virginia to Washington's starving army in Pennsylvania. (See Botta's History.) These agricultural products were created by the labor of savages naturally so indolent in their native Africa, as to prefer to live on ant eggs and caterpillars rather than labor for a subsistence; but for years in succession they continued to labor in the midst of their masters' enemies—dropping their hoes when they saw the red-coat, running to tell their mistress and to conduct her and the children through by-paths to avoid the British troopers, and when the enemy were out of sight, returning to their work again. The sole cause of their industry and fidelity is due to spiritual influence of the white race over the black.

The empire of the white man's will over the prognathous race is not absolute, however. It cannot force exercise beyond a certain speed; neither the will nor physical force can drive negroes, for a number of days in succession, beyoud a very moderate daily labor—about one-third less than what the white man voluntarily imposes on himself. If force be used to make them do more, they invariably do less and less, until they fall into a state of impassivity, in which they are more plague than profit—worthless as laborers, insensible and indifferent to punishment, or even to life; or, in other words, they fall into the disease which I have named Dysesthæsia Ethiopica, characterized by hebetude of mind and insensibility of body, caused by overworking and bad treatment. Some knowledge of the ethnology of the prognathous race is absolutely necessary for the prevention and cure of this malady in all its various forms and stages. Dirt eating, or Cachexia Africana, is another disease, like Dysesthæsia Ethiodica, growing out of ethnical elements peculiar to the prognathous race. The ethnical elements assimilating the negro to the mule, when compared with the horse, although giving rise to the last named disease, are of vast importance to the prognathous race, because they guarantee to that race an ample protection against the abuses of arbitrary power. A white man, like a blooded horse, can be worked to death. Not so the negro, whose ethnical elements, like the mule restrict the limits of arbitrary power over him. Among the four millions of the prognathous race in the United States, it will be difficult, if not impossible, to find a single individual negro, whom the white man, armed with arbitrary power, has ever been able to make him hurt himself at work. It is beyond the power of the white man to drive the negro into those long continued and excessive muscular exertions, such as the white laborers of Europe often impose upon themselves to satisfy a greedy boss, under fear of losing their places and thereby starving themselves and families. Throughout England, nothing is more common than decrepitude, premature old age, and a frightful list of diseases caused by long continued and excessive muscular exertion. Whereas, all America can scarcely furnish an example of the kind among the prognathous race. The white men of America have peformed many prodigies, but they have never yet been able to make a negro overwork himself.

There are other elements peculiar to the Nigritian, on which the disease, called negro consumption, or Cachexia Aicana, depends. But these belong to that class which subject the negro to the white man's spiritual empire over him. When that spiritual empire is not maintained in all its entirety, or in other words, when the negro is badly governed, he is apt to fall under the spiritual influence of the artful and designing of his own color and Cachexia Africana, or consumption, is the consequence. Better throw medicine to the dogs, than give it to a negro patient impressed with the belief that he has walked over poison specially laid for him, or been in some other way tricked or conjured. He will surely die, unless treated in accordance with his ethnological peculiarities, and the hallucination expelled.

There never has been an insurrection of the prognathous race against their masters; and from the nature of the ethnical elements of that race, there never can be. Hayti is no exception, it will be seen, when the true history of the so-called insurrection of that island is written. There have been neigborhood disturbances and blood-shed caused by fanaticism, and by mischevious white men getting among them and infusing their will into them, or mesmerizing them. But fortunately, there is an ethnological law of their nature which estops the evil influence of such characters by limiting their influence strictly to personal acquaintances. The prognathous tribes in every place and country are jealous and suspicious of all strangers, black or white and have ever been so.

Prior to the Emancipation Act in the British West Indies, the famous Exeter Hall Junto sent out a number of emissaries of the East India Company to Jamaica, in the garb of missionaries. After remaining a year or two in the assumed character of Christian ministers, they began to preach insurrectionary doctrines, and caused a number of so-called insurrections to break out simultaneously in different parts of the island. The insurgents in every neighborhood were confined to the personal acquaintances of the Exeter Hall miscreants, who succeeded in infusing their will only into those who had listened to their incendiary harangues.

This was proved upon them by the genuine missionaries, who had long been on the island, and had gathered into their various churches a vast number of converts. For, in no instance, did a single convert, or any other negro, join in the numerous insurrectionary movement, who had not been personally addressed by the wolves in sheep's clothing. The Christian missionaries, particularly the Methodist, Baptist, Moravians and Catholics, were very exact in collecting the evidences of this most important ethnological truth, in consequence of some of the planters, at the first outbreak, having confounded them with the Exeter Hall incendiaries. The planters finally left the Christian missionaries and their flocks undisturbed, but proceeded to expel the false missionaries; to hang their converts, and to burn down their chapels. The event proved that they were wrong in not hanging the white incendiaries; because they went home to England, preached a crusade—traveling all over the United Kingdom—proclaiming, as they went, that they had left God's houses in flames throughout Jamaica, and God's people hanging like dogs from the trees in that sinful island. This so inflamed public sentiment in Great Britain against the planters, as to unite all parties in loud calls for the immediate passage of the Emancipation act. There is good reason to believe that the English Ministry, in view of the probable effect of that measure on the United States, and the encouragement it would afford to the culture of sugar and other tropical products in the East Indies and Mauritius, had previously determined to make negro freedom a leading measure in British policy, well knowing that its effect would be to Africanize the sugar and cotton growing regions of America. The ethnology of the prognathous race does not stop at proving that subordination to the white race is its normal condition. It goes further, and proves that social and political equality is abnormal to it, whether educated or not. Neither negroes nor mulattoes know how to use power when given to them. They always use it capriciously and tyranically.—Tschudi, a Swiss naturalist, (see Tschudi's Travels in Peru, London, 1848,) says, "that in Lima and Peru generally, the free negroes are a plague to society. Dishonesty seems to be a part of their very nature.—Free born negroes, admitted into the houses of wealthy families, and who have received in early life, a good education, and treated with kindness and liberality do not differ from their uneducated brethren."

Tschudi is mistaken in supposing that dishonesty is too deeply rooted in the negro character to be removed. They are dishonest when in the abnormal condition without a master. They are also dishonest when in a state of subordination, called slavery, badly provided for and not properly disciplined and governed. But when properly disciplined, instructed and governed, and their animal wants provided for, it would be difficult to find a more honest, faithful and trustworthy people than they are. When made contented and happy, as they always should be, they reflect their master in their thoughts morals and religion, or at least they are desirous of being like him. They imitate him in every thing, as far as their imitative faculties, which are very strong, will carry them. They take a pride in his wealth, or in anything which distinguishes him, as if they formed a part of himself, as they really do, being under the influence of his will, and in some measure assimilated, in their spiritual nature, to him—loving him with all the warm and devoted affection which children manifest to their parents. He is sure of their love and friendship, although all the world may forsake him. But to create and maintain this happy relation, he must govern them with strict reference to their ethnological peculiarities. He must treat them as inferiors, not as equals, as they are not satisfied with equality, and will despise a master who attempts to raise any one or more of them to an equality whith himself; because they become jealous and suspicious that their masters's favorites will exercise a sinister influence over him against them. Impartiality of treatment in every particular, down to a hat or pair of shoes, is what they all regard as one of their dearest rights. Hence, any special favors or gifts to one, is an offence to all the rest. They also regard as a right, when punished, not to be punished in anger, but with cool deliberation. They will run from an angry or enraged master or overseer, armed with a gun or a pistol. They regard all overseers who come into the field armed with deadly weapons as cowards, and all cowards have great difficulty in governing them. It is not physical force which keeps them in subjection, but the spiritual force of the white man's will. One unarmed brave man can manage a thousand by the moral force of his will alone, much better than an hundred cowards with guns in their hands. They also require as a right when punished, to be punished with a switch or a whip, and not with stick or the fist. In this particular the ethnical law of their nature is different from all other races of men. It is exactly the reverse of that of the American Indian. The Indian will murder any man who strikes him with a switch, a cowhide or a whip twenty years afterwards, if he gets an opportunity; but readily forgives and forgets blows, however severe, inflicted on him with the fist, a cudgel or a tomahawk. A remarkable ethnological peculiarity of the prognathous race is, that any deserved punishment, inflicted on them with a switch, cowhide or whip, puts them into a good humor with themselves and the executioner of the punishment, provided he manifest satisfaction by regarding the offence as atoned for.

The negro requires government in every thing, the most minute. The Indian, on the contrary, submits to government in nothing whatever. Mr. Jefferson was the first to notice this ethnical law of the red man. [See his letter to Gilmer, June 7, 1816, vol. iv. page 279; *Jefferson's Correspondence.*] "Every man with them," (the Indians,) says Mr. Jefferson, "is perfectly free to follow his own inclinations; but if, in doing this, he violates the rights of another, he is punished by the dis-esteem of society or tomahawked. Their leaders conduct them by the influence of their character only; and they follow or not, as they please, him, of whose character for wisdom or war, they have the highest opinion; but of all things, they least think of subjecting themselves to the will of one man." Whereas, the black man requires government even in his meat and drink, his clothing and hours of repose. Unless under the government of one man to prescribe rules of conduct to guide him, he will eat too much meat, and not enough of bread and vegetables; he will not dress to suit the seasons, or kind of labor he is engaged in, nor retire to rest in due time to get sufficient sleep, but sit up and doze by the fire nearly all night. Nor will the women undress the children and put them regularly to bed. Nature is no law unto them. They let their children suffer and die, or unmerciful.y abuse them, unless the white man or woman prescribe rules in the nursery for them to go by. Whenever the white women superintend the nursery, whether the climate be cold or hot, they increase faster than any other people on the globe; but on large plantations, remote from her influence, the negro population invariably diminishes, unless the overseer take upon himself those duties in the lying-in and nursery department, which on small estates are attended to by the mistress. She often sits up at night with sick children, and administers to their wants, when their own mothers are nodding by them, and would be sound asleep if it were not for her presence. The care that white women bestow on the nursery, is one of the principal causes why three hundred thousand Africans, originally imported into the territory of the United States, have increased to four millions; while in the British West Indies the number imported exceed, by several mil-tions, the actual population. It is also the cause, why the small proprietors of negro property in Maryland, Virginia, Kentucky and Missouri are able to supply the loss on the large Southern plantations, which are cut off from the happy influence of the presiding genius over civilization, morality and population—the white woman.

The prognathous race require government also in their religious exercises, or they degenerate into fanatical saturnalia. A discreet white man or woman should always be present to regulate their religious meetings.

Here the investigation into the ethnology of the prognathous race must close, at least for the present, leaving the most interesting part, Fetechism, the indigenous religion of the African tribes, untouched. It is the key to the negro character—which is difficult to learn from mere experience. Those who are not accustomed to them have great trouble and difficulty in managing negroes; and in consequence thereof, treat them badly. If their ethnology was better and more generally understood, their value would be greatly increased and their condition, as a laboring class, would be more enviable, compared to the European peasants, than it already is.—*N. O. Med. & Surg. Journal.*

